# A neural field model using advanced anatomical connectivity information

**DOI:** 10.1186/1471-2202-12-S1-P174

**Published:** 2011-07-18

**Authors:** Christopher Koch, Manh Nguyen Trong, Andreas Spiegler, Thomas R Knösche

**Affiliations:** 1 Max Planck Institute for Human Cognitive and Brain Sciences, Leipzig, Germany; 2 Institute for Biomedical Engineering and Informatics, Ilmenau University of Technology, Ilmenau, Germany

## 

We propose a mathematical framework for a neural field model that can accommodate empirical information on connectivity strength between different parts of the brain, and axonal caliber information of these connections. Furthermore, we use integro-differential equations to describe the mean dynamics (i.e., firing rate and mean membrane potential) [1]. We demonstrate the framework at the example of the rat brain.

**Figure 1:**
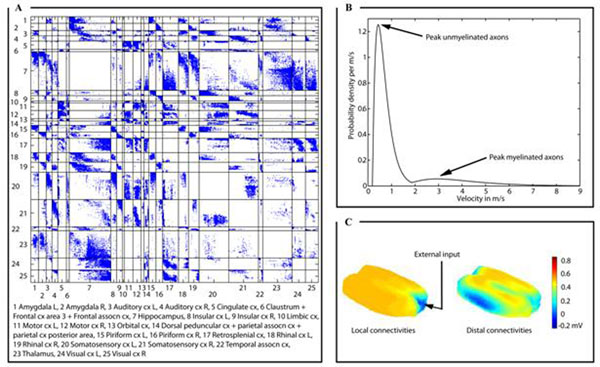
**A.** Connectome **B.** Velocity probability density **C.** Electrocorticogram

Here, we specify the propagation velocity distributions by a linear relationship using empirical, position-variant, axonal diameter distributions of myelinated and unmyelinated callosal axons [2]. We approximate the experimentally estimated histograms of axonal diameters using alpha functions. By interpolating these alpha functions in space, weighted by the fiber densities of the myelinated and unmyelinated axons, we compute the velocity probability density (see Figure 1B). Diffusion tensor imaging is used to reconstruct axonal projections through the white matter. We use an atlas-based parcellation of the rat brain [3] to allocate the reconstructed projections to specific brain regions, yielding a connectome (see Figure 1A). The structures that are most strongly interconnected are the hippocampus, the thalamus, the motor and the sensory cortices. A simulation of the electrocorticogram demonstrates the impact of distal over local connections on brain function (see Figure 1C).
